# Study on risk stratification and treatment strategy of blood indicators in patients with moderate risk of GIST

**DOI:** 10.3389/fonc.2026.1742064

**Published:** 2026-04-29

**Authors:** Xincheng Su, Jinhu Chen, Zhiming Cai, Lv Lin, Zhenrong Yang, Tao Lin, Weibin Song, Xinyu Chen, Zihan Lin, Yongjian Zhou

**Affiliations:** 1 Department of Gastric Surgery, Fujian Medical University Union Hospital, Fuzhou, China; 2 Department of Gastrointestinal Surgery, The First Affiliated Hospital of Fujian Medical University, Fuzhou, China

**Keywords:** GIST, inflammatory biomarkers, intermediate-risk stratification, machine learning, prognosis

## Abstract

**Background:**

Intermediate-risk gastrointestinal stromal tumor (GIST) patients exhibit marked prognostic heterogeneity. The traditional NIH risk classification often results in undertreatment of latent high-risk patients and overtreatment of truly low-risk ones. This study aimed to develop an interpretable machine learning model integrating hematologic inflammatory markers to achieve precise risk re-stratification and optimize adjuvant therapy strategies for intermediate-risk patients.

**Methods:**

Primary GIST patients were retrospectively enrolled. LASSO regression was applied to select key features from eight inflammatory markers (including NLR, PLR, and SII). A random survival forest model was then constructed, followed by 5-fold cross-validation. SHAP values were used to interpret feature contributions, and Kaplan–Meier survival analysis was conducted to evaluate stratification performance.

**Results:**

LASSO regression identified seven inflammatory markers, among which PLR, SII, and PIV were the top three key variables. The optimal random survival forest model (five-feature model) achieved an AUC of 0.777, with an internally validated mean AUC of 0.782 (95% CI: 0.679–0.878) and an out-of-bag (OOB) error of 0.124. SHAP analysis revealed that PLR, NLR, and PAR were the major contributors to model prediction. The model effectively stratified intermediate-risk patients into “intermediate–high-risk” and “intermediate–low-risk” subgroups with significantly different survival outcomes (p<0.0001).

**Conclusion:**

This study represents the first construction of an interpretable predictive model integrating blood-based inflammatory markers with machine learning algorithms. The model accurately identifies occult high-risk individuals among patients with intermediate-risk GIST, thereby providing exploratory evidence and a foundation for hypothesis generation for future individualized management strategies.

## 1 Introduction

Gastrointestinal stromal tumors (GISTs), the most prevalent mesenchymal neoplasms of the gastrointestinal tract, are presumed to arise from the interstitial cells of Cajal (or their precursors). Although many localized GISTs can be managed successfully by surgical excision, these tumors inherently harbor malignant potential, and their aggressiveness is conventionally stratified by tumor size, mitotic index (mitotic count), and anatomical site (1). In current clinical consensus, three clinicopathologic parameters — tumor size, mitotic index, and anatomical location (e.g. gastric vs non-gastric) — represent the principal determinants of malignant potential and recurrence risk in GIST. Although the 2008 modified NIH risk stratification is widely utilized to distinguish low- and high-risk GIST cases, it exhibits marked limitations in predicting outcomes within the intermediate-risk category, which remains heterogeneous.

Evidence indicates that 5-year recurrence-free survival (RFS) rates vary substantially among patients classified as intermediate risk (2). For instance, a study applying the Genomic Grade Index (GGI) to AFIP-designated intermediate/high-risk GIST patients subdivided them into “low-risk” and “high-risk” strata, yielding 5-year RFS rates of approximately 73% vs. 35%, respectively — implicating a subset of “occult high-risk” patients within the intermediate tier (3). Furthermore, for intermediate-risk patients considered for adjuvant imatinib therapy, a dilemma persists: under-treatment of latent high-risk individuals on the one hand, and overtreatment of bona fide low-risk patients on the other. The insufficiency of existing risk stratification systems to reliably distinguish these subpopulations underscores the urgent need for more refined prognostic models.

Similar to radiomics, machine learning models based on blood inflammatory markers can also capture tumor-associated systemic inflammatory responses, providing a non-invasive approach for tumor prognosis assessment (4). Hematologic biomarkers reflecting systemic inflammatory burden — including platelet-to-lymphocyte ratio (PLR) (5), neutrophil-to-platelet ratio (NPR) (6), neutrophil-to-lymphocyte ratio (NLR) (7), platelet-to-albumin ratio (PAR) (8), lymphocyte-to-monocyte ratio (LMR) (9), pan-immune inflammation value (PIV) (10), systemic immune-inflammation index (SII) (11)and systemic inflammation response index (SIRI) (12)— have been proposed as potential proxies of the host immune–inflammatory milieu in oncologic settings. A substantial body of literature has established that such inflammatory indices frequently correlate with prognosis across diverse malignancies. For instance, in colorectal cancer, elevated PLR and SII have been consistently linked to inferior outcomes, indicating that systemic inflammatory status may inform disease stratification and clinical decision making (13). In the context of GIST, several studies have more thoroughly evaluated NLR, PLR, SII, and LMR as predictive indices, demonstrating significant associations with recurrence risk (14, 15). For example, a meta-analysis in GIST populations reported that elevated PLR and NLR were significantly associated with inferior disease-free survival (DFS)/RFS (16). In contrast, parameters such as PAR, PIV, and SIRI remain relatively underexplored in GIST, and their prognostic significance is still largely undefined. Therefore, this study incorporated these eight most representative and clinically accessible inflammatory composite indices as candidate features.

This study collected clinical data from 661 multi-center GIST patients and constructed a predictive model based on hematological parameters using machine learning methods, followed by internal validation via 5 × 200-fold cross-validation. Risk stratification analysis was performed based on the model outputs, aiming to provide a refined tool for adjuvant treatment decision-making in intermediate-risk GIST patients who have undergone radical resection. Overall, this study empirically demonstrates that modeling hematological indicators with machine learning techniques can offer an effective approach for early recurrence warning and precision medicine intervention in intermediate-risk GIST patients.

## 2 Materials and methods

### 2.1 patients and data

**Figure 1:**
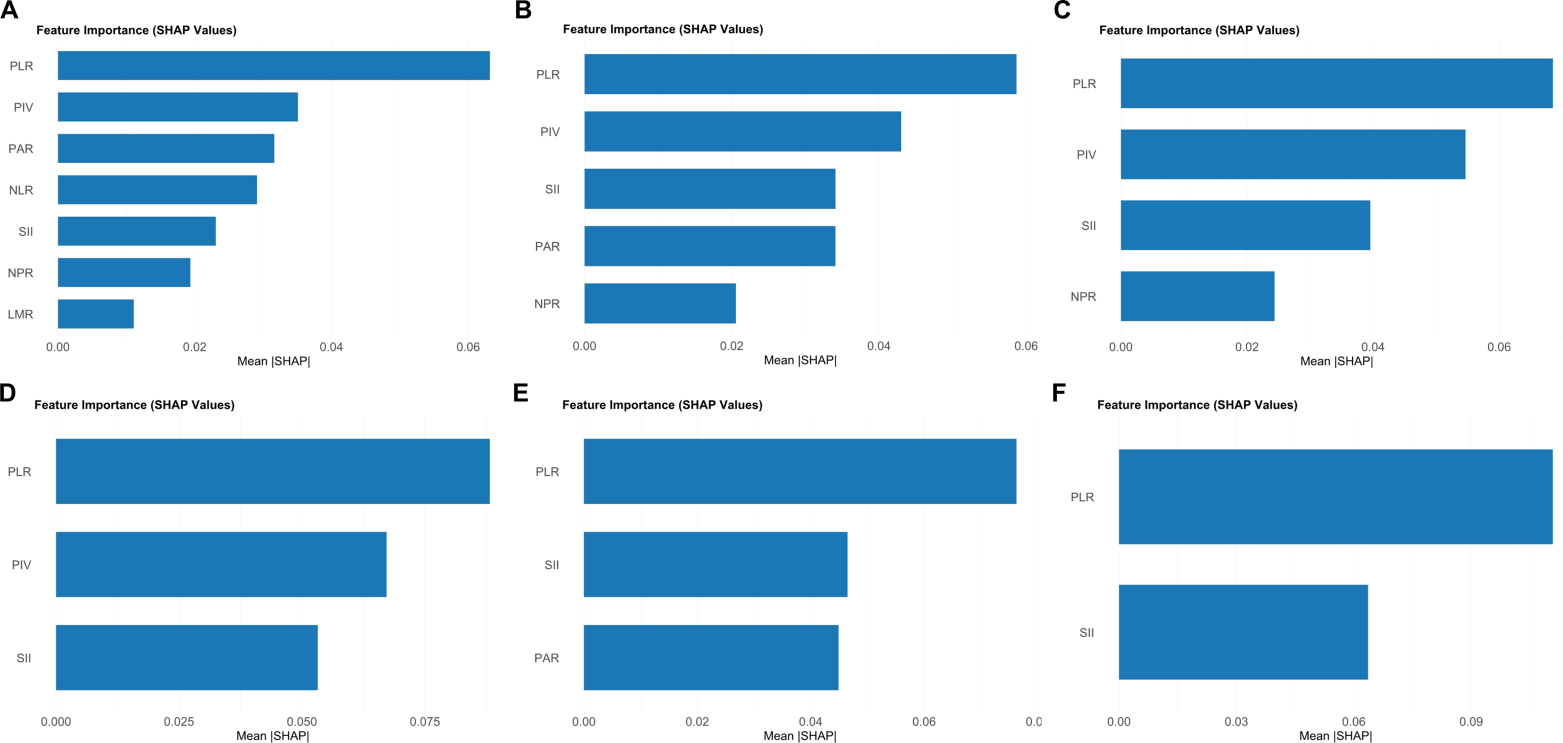
Schematic overview of the study.

This study retrospectively collected data from 813 patients with gastrointestinal stromal tumors (GIST) who were admitted to the Affiliated Union Hospital of Fujian Medical University and Fujian Provincial Cancer Hospital between January 2010 and December 2022. After screening based on the inclusion and exclusion criteria, a total of 660 patients were ultimately included in the analysis. The study flow chart is shown in **Figure 1**.

Screening was conducted according to specific criteria. Inclusion criteria: (1) Confirmed diagnosis of GIST by postoperative pathological examination; (2) No evidence of distant metastasis on preoperative imaging studies (including chest X-ray, abdominal ultrasound, and CT); (3) Underwent radical surgical resection with R0 or R1 margins; (4) Age ≥ 18 years at diagnosis; (5)Classified as intermediate risk according to the 2008 modified NIH criteria. According to the 2008 modified NIH criteria, intermediate-risk GIST was defined as follows: for gastric GIST, meeting any of the following criteria—mitotic count of 6–10 per 50 HPF with a maximum tumor diameter of 2–5 cm, or mitotic count <5 per 50 HPF with a maximum tumor diameter of 5.1–10 cm; for non-gastric GIST, defined as mitotic count of 6–10 per 50 HPF with a maximum tumor diameter ≤2 cm. Exclusion criteria: (1) Preoperative receipt of tyrosine kinase inhibitor therapy; (2) Incomplete clinical or pathological data; (3) Concurrent other malignancies; (4) Death due to non-GIST-related causes or loss to follow-up within one month post-surgery. To ensure consistency in risk classification, the maximum tumor diameter and mitotic count for all cases were reviewed by senior pathologists from each center according to the 2008 modified NIH criteria; in cases of discrepancy, consensus was reached through consultation.

This study was approved by the Ethics Committee of the Affiliated Union Hospital of Fujian Medical University and conducted in accordance with the Declaration of Helsinki (Ethics Code: 2025KY604). Informed consent was waived due to the retrospective nature of the study. To ensure participant privacy, we anonymized personal information using unique identifiers and stored personal data separately from public data.

### 2.2 Data preprocessing

The missing rate for all hematological indicators and covariates was less than 5%. A complete case analysis was adopted, whereby any patient with missing data was excluded. Peripheral blood samples were collected within three days prior to surgery. Patients with acute infection, active inflammation, or those receiving hormone therapy within two weeks before surgery were excluded. The calculation of peripheral blood indices included: a. Platelet-to-Lymphocyte Ratio (PLR) = Absolute Platelet Count/Absolute Lymphocyte Count; b. Neutrophil-to-Platelet Ratio (NPR) = Absolute Neutrophil Count/Absolute Platelet Count; c. Neutrophil-to-Lymphocyte Ratio (NLR) = Absolute Neutrophil Count/Absolute Lymphocyte Count; d. Platelet-to-Albumin Ratio (PAR) = Absolute Platelet Count/Albumin Count; e. Lymphocyte-to-Monocyte Ratio (LMR) = Absolute Lymphocyte Count/Absolute Monocyte Count; f. Systemic Immune-Inflammation Index (SII) = Absolute Platelet Count × Absolute Neutrophil Count/Absolute Lymphocyte Count; g. Pan-Immune-Inflammation Value (PIV) = Absolute Neutrophil Count × Absolute Platelet Count × Absolute Monocyte Count/Absolute Lymphocyte Count; h. Systemic Inflammation Response Index (SIRI) = Absolute Neutrophil Count × Absolute Monocyte Count/Absolute Lymphocyte Count.

### 2.3 Diagnostic prediction model

This study constructed a two-stage machine learning-based prediction model for the stratification of intermediate-risk patients. First, Lasso regression (glmnet package) was employed for feature selection from eight candidate inflammatory markers (PLR, SII, PIV, NPR, PAR, LMR, NLR, SIRI). The optimal lambda value (lambda.min) was determined via 10-fold cross-validation, and features with non-zero coefficients were retained for subsequent modeling. Subsequently, a Random Forest algorithm (ranger package) was used to build the prediction model, with the number of trees (num.trees) set to 500 and the splitting rule (splitrule) as the Gini index. Key parameters were optimized through grid search: the number of features randomly selected at each split (mtry) was optimized within a range from 1 to the total number of features, and the minimum node size (min.node.size) was optimized among 1, 5, and 10. Model performance was evaluated using 5-fold cross-validation, with the Area Under the Curve (AUC) as the optimization metric.

Model interpretability analysis was performed using the SHAP (SHapley Additive exPlanations) method. Feature contributions were calculated using the fastshap package, with visualizations including feature importance ranking plots and beeswarm plots. Finally, based on the optimal cutoff value determined by the Youden index applied to the model’s predicted probabilities, intermediate-risk patients were stratified into two subgroups: “MR-L” and “MR-H”. All analyses were performed with a fixed random seed (seed=123) to ensure reproducibility.

### 2.4 Internal validation

Given the limited sample size, an independent validation cohort was not established. The model’s generalization performance was assessed using 5 × 200-fold cross-validation; the mean AUC and its 95% confidence interval (calculated based on 1000 bootstrap resampling results) are reported.

### 2.5 Statistical analysis

Statistical analyses were performed using R version 4.5.1. Categorical variables were presented as frequencies (percentages), and continuous variables as medians with interquartile ranges.

Model performance was evaluated using the following approaches: (1) Time-dependent AUC calculation: The 5-year time-dependent area under the ROC curve (AUC) was calculated using the timeROC package, with censored data handled via the nearest neighbor estimation method. To assess model stability, the AUC was repeatedly computed for each fold of the 5 × 200-fold cross-validation using the survivalROC package with the nearest neighbor estimation; (2) Classification performance metrics calculation: Based on the optimal cut-off value, accuracy, sensitivity, specificity, F1-score, positive predictive value (PPV), and negative predictive value (NPV) were calculated using the pROC package. The optimal cut-off value for predicted probabilities was determined using the Youden index. To assess the stability of this cut-off, the procedure was repeated within the training set of each cross-validation fold, and the mean and standard deviation of the cut-off values were calculated.

Survival analysis was conducted using the Kaplan-Meier method to compare recurrence-free survival (RFS) among groups, with P-values calculated by the Log-rank test. Survival curves were truncated at a 60-month follow-up period. SHAP values were computed with 50 Monte Carlo simulations to stabilize the estimates.

All statistical tests were two-sided, and a P-value < 0.05 was considered statistically significant. Visualizations were generated using the ggplot2 and survminer packages, and the reporting of results adhered to the TRIPOD (Transparent Reporting of a multivariable prediction model for Individual Prognosis Or Diagnosis) statement guidelines.

## 3 Results

### 3.1 Patient characteristics

A total of 661 patients were included in this study between January 2010 and December 2022. Key demographic characteristics and treatment details are summarized in **Table 1**. Among patients of all risk levels, the median age was 58.0 years (interquartile range [IQR] 51.0-66.0), and 51.3% were male. High-risk patients accounted for 46.9%, intermediate-risk for 23.3%, and low/very low-risk for 29.7%. The tumor location was gastric in 73.2% of cases and non-gastric in 26.8%. Within the intermediate-risk patient subgroup, the median age was 58.0 years (IQR 52.0-67.0), and 53.9% were male. The tumor location was gastric in 94.8% and non-gastric in 5.2%. The classification of intermediate-risk patients strictly followed the 2008 modified NIH criteria.

**Table 1:** The characteristics and clinical data of participants.

Variables	All (%)	Medium risk (%)	P value
Total	661	154	
Age, years	58 (51.0-66.0)	58 (52.0-67.0)	0.234
Sex			0.567
Male	339 (51.3)	83 (53.9)	
Female	321 (48.7)	71 (46.1)	
Hypertension			0.189
yes	197 (29.8)	53 (34.4)	
no	463 (70.2)	101 (65.6)	
Diabetes			0.312
yes	91 (13.8)	17 (11.0)	
no	569 (86.2)	137 (89.0)	
NIH			<0.001
Very low/low risk	310 (46.9)	/	
Medium risk	154 (23.3)		
High risk	196 (29.7)		
Maximum tumor diameter	5.6 (3.4-6.6)	5.7 (3.1-6)	0.874
Mitotic count	4.8 (1-5)	3.6 (1-5)	0.015
Site			<0.001
Stomach	484 (73.2)	146 (94.8)	
Non-stomach	176 (26.8)	8 (5.2)	
Type of genetic mutation			0.452
Kit 11 mutation	465 (70.3)	105 (68.2)	
Kit 9 mutation	105 (15.9)	24 (15.6)	
PDGFR-α	29 (4.4)	9 (5.8)	
Wild type	51 (7.7)	14 (9.1)	
Others	10 (1.5)	2 (1.3)	
TKI used			<0.001
yes	253 (38.3)	90 (58.4)	
no	407 (61.7)	64 (41.6)	

### 3.2 Prognostic factor analysis

In the entire patient cohort, univariate Cox regression analysis (**Table 2**) revealed that maximum tumor diameter (MAXDiameter, HR = 1.10, 95% CI 1.10-1.20, p<0.001), mitotic count (MitosesAccounts, HR = 1.00, 95% CI 1.00-1.00, p<0.001), and all inflammatory markers except NPR (PAR, PIV, SIRI, SII, NLR, PLR, LMR; all p<0.001) were significantly associated with RFS. A history of diabetes demonstrated a protective effect (HR = 0.35, 95% CI 0.16-0.74, p=0.006). Demographic characteristics such as age, sex, and hypertension showed no statistical significance (all p>0.05). Multivariate analysis further identified the following independent prognostic factors: 1. Clinicopathological factors: maximum tumor diameter (HR = 1.10, 95% CI 1.00-1.10, p<0.001) and mitotic count (HR = 1.00, 95% CI 1.00-1.00, p=0.037). 2. Inflammatory markers: Neutrophil-to-Lymphocyte Ratio (NLR, HR = 1.30, 95% CI 1.10-1.40, p<0.001) and Systemic Immune-Inflammation Index (SII, HR = 1.00, 95% CI 1.00-1.00, p=0.013). Metabolic factor: history of diabetes (HR = 0.44, 95% CI 0.20-0.96, p=0.038). Notably, inflammatory markers significant in univariate analysis, such as PAR and PIV, lost their independence in the multivariate model (all p>0.05), suggesting their prognostic value might be overshadowed by stronger predictors like NLR.

**Table 2:** Univariate and multivariate analysis (All patients).

Variate	Univariate analysis	P-value	Multivariate analysis	P-value
	HR (95% CI)		HR (95% CI)	
Age	1.00 (0.99–1.00)	0.270	-	-
Gender	1.00 (0.73–1.50)	0.850	-	-
Hypertension	0.89 (0.60–1.30)	0.540	-	-
Diabetes	0.35 (0.16–0.74)	0.006	0.44 (0.20–0.96)	0.038
MAXDiameter	1.10 (1.10–1.20)	<0.001	1.10 (1.00–1.10)	<0.001
MitosesAccounts	1.00 (1.00–1.00)	<0.001	1.00 (1.00–1.00)	0.037
PAR	1.20 (1.20–1.20)	<0.001	1.00 (0.93–1.10)	0.780
PIV	1.00 (1.00–1.00)	<0.001	1.00 (1.00–1.00)	0.180
SIRI	1.10 (1.00–1.10)	<0.001	0.83 (0.65–1.10)	0.150
SII	1.00 (1.00–1.00)	<0.001	1.00 (1.00–1.00)	0.013
NLR	1.10 (1.10–1.20)	<0.001	1.30 (1.10–1.40)	<0.001
PLR	1.00 (1.00–1.00)	<0.001	1.00 (1.00–1.00)	0.260
LMR	0.78 (0.70–0.86)	<0.001	1.00 (0.96–1.10)	0.440
NPR	1.00 (1.00–1.00)	0.710	-	-

In the intermediate-risk patient subgroup analysis (**Table 3**), univariate Cox regression showed: 1. Tumor characteristics: maximum tumor diameter (MAXDiameter) was significantly associated with poor prognosis (HR = 1.30, 95% CI 1.00-1.50, p=0.032), while mitotic count (MitosesAccounts) showed a protective effect (HR = 0.82, 95% CI 0.69-0.99, p=0.037). 2. Inflammatory markers: Only Platelet-to-Albumin Ratio (PAR, HR = 1.20, 95% CI 1.10-1.40, p<0.001), Systemic Immune-Inflammation Index (SII, HR = 1.00, 95% CI 1.00-1.00, p<0.001), Neutrophil-to-Lymphocyte Ratio (NLR, HR = 1.40, 95% CI 1.10-1.60, p<0.001), and Platelet-to-Lymphocyte Ratio (PLR, HR = 1.00, 95% CI 1.00-1.00, p<0.001) were significantly associated with RFS. Demographic features like age, sex, hypertension, and inflammatory markers such as NPR and LMR showed no statistical significance (all p>0.05). Multivariate analysis results indicated that after multivariable adjustment, none of the indicators significant in the univariate analysis retained their independence: maximum tumor diameter (HR = 1.00, 95% CI 0.79-1.40, p=0.750), mitotic count (HR = 0.85, 95% CI 0.69-1.00, p=0.130), and inflammatory markers (PAR, SII, NLR, etc., all p>0.05).

**Table 3:** Univariate and multivariate analysis (Medium risk patients).

Variable	Univariate analysis	P-value	Multivariate analysis	
	HR (95% CI)		HR (95% CI)	P-value
Age	1.00 (0.97-1.00)	0.690	-	-
Gender	1.10 (0.53-2.20)	0.810	-	-
Hypertension	1.50 (0.73-3.10)	0.260	-	-
Diabetes	0.85 (0.26-2.80)	0.780	-	-
MAXDiameter	1.30 (1.00-1.50)	0.032	1.00 (0.79-1.40)	0.750
MitosesAccounts	0.82 (0.69-0.99)	0.037	0.85 (0.69-1.00)	0.130
PAR	1.20 (1.10-1.40)	<0.001	0.98 (0.77-1.20)	0.850
PIV	1.00 (1.00-1.00)	0.004	1.00 (1.00-1.00)	0.730
SIRI	1.30 (0.96-1.80)	0.085	0.82 (0.18-3.80)	0.800
SII	1.00 (1.00-1.00)	<0.001	1.00 (0.97-1.00)	0.780
NLR	1.40 (1.10-1.60)	<0.001	1.20 (0.63-2.30)	0.580
PLR	1.00 (1.00-1.00)	<0.001	1.00 (1.00-1.00)	0.110
LMR	0.92 (0.75-1.10)	0.440	-	-
NPR	1.00 (0.99-1.00)	0.430	-	-

### 3.3 Feature selection and predictive performance

In GIST patients from all tumor locations, when all 8 features were input, LASSO regression selected 7 features, ranked by importance as: PLR, PIV, PAR, NLR, SII, NPR, LMR. Progressively removing features starting from the least important resulted in different models. In the gastric GIST subgroup, when all 8 features were input, LASSO regression selected only 3 features, ranked by importance as: PLR, SII, PAR. When the number of input features was reduced, dropping to 4 features resulted in only 2 remaining features: PLR and SII.

Through systematic comparison of model performance with different numbers of features (**Table 4**), we made the following findings.

**Table 4:** Comparison of model performance.

Model	AUC	Accuracy	Sensitivity	Specificity	PPV	NPV	OOB
All patients-7 indicators	0.76	0.842	0.417	0.941	0.625	0.873	0.129
All patients-5 indicators	0.777	0.84	0.406	0.941	0.619	0.871	0.124
All patients-4 indicators	0.758	0.84	0.396	0.944	0.623	0.87	0.123
All patients-3 indicators	0.762	0.832	0.406	0.932	0.582	0.87	0.122
Gastric patients-3 indicators	0.706	0.882	0.25	0.976	0.611	0.897	0.105
Gastric patients-2 indicators	0.687	0.879	0.25	0.973	0.579	0.897	0.11

(1) In GIST patients from all locations, the 5-feature model (“PLR”, “SII”, “PIV”, “NPR”, “PAR”) demonstrated the best comprehensive performance (AUC = 0.777). While maintaining high specificity (0.941), it achieved a relatively optimal sensitivity (0.406), with Positive Predictive Value (PPV = 0.619) and Negative Predictive Value (NPV = 0.871) reaching clinically applicable standards. The Out-Of-Bag (OOB) error for all models remained stable within the range of 0.122-0.129. The OOB error for the best model (the 5-feature model) was 0.124, indicating good generalization ability. This suggests that more features are not necessarily better; the 5-feature combination achieved the highest AUC and a low OOB error. Given the limited sample size in this study and the absence of an independent validation set, internal validation using a strategy of 5-fold cross-validation repeated 200 times was performed based on the model built with the 5-feature combination. The results showed a mean AUC of 0.782 (95% CI: 0.679–0.878) for the model, indicating good discriminatory ability.

(2) Analysis in the gastric GIST subgroup revealed characteristic differences compared to the overall model. Both the Lasso-selected 3-feature model (PLR, SII, PAR) and the 2-feature model (PLR, SII) demonstrated very high specificity (0.976 and 0.973, respectively), but relatively low sensitivity (both 0.25). Accuracy and NPV were superior to the overall model, whereas AUC, PPV, and OOB error were comparatively inferior.

### 3.4 Model interpretation

**Figure 2:**
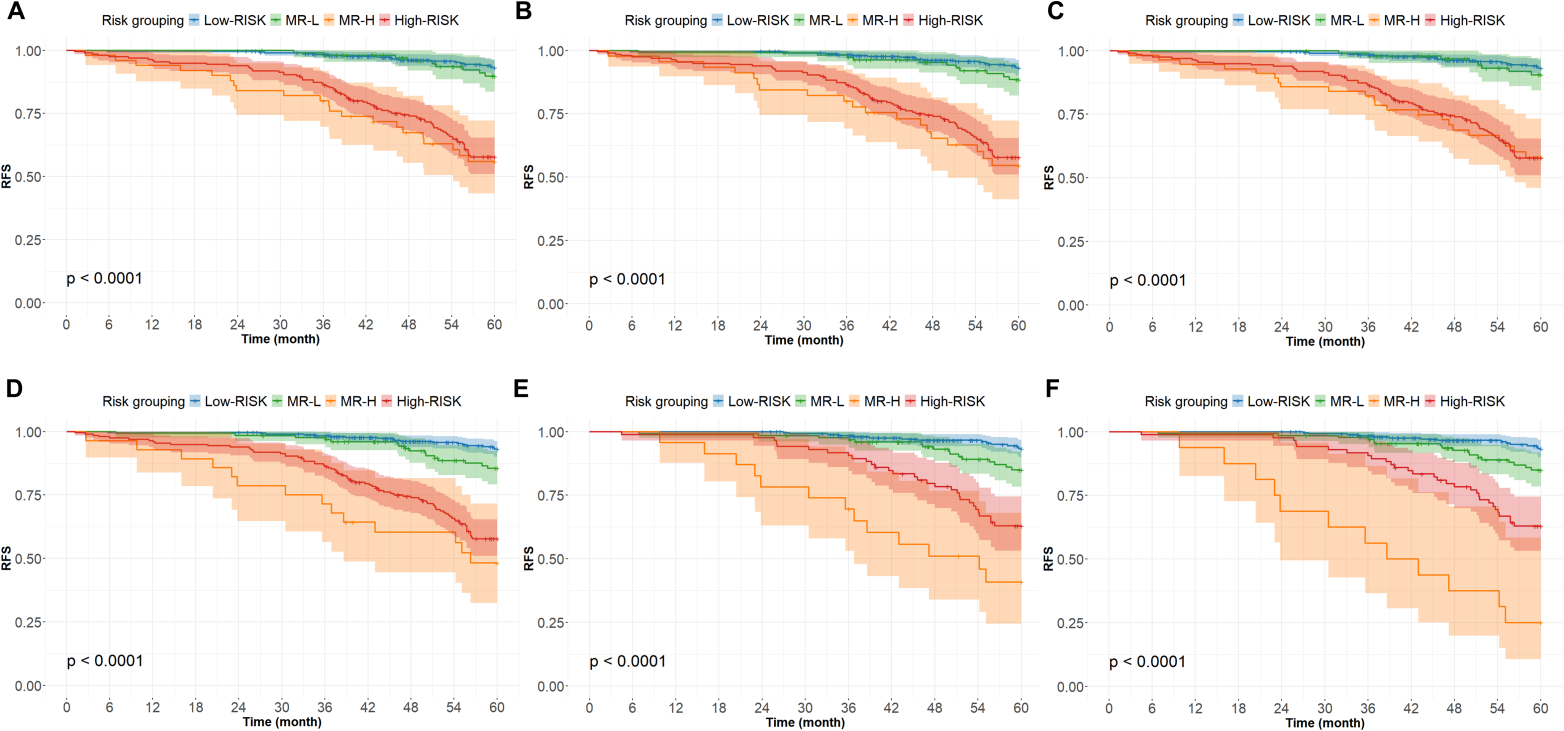
Ranking of predictive importance of inflammatory markers under different feature combinations. **(A)** Screening results of 8 initial characteristics in the overall patient cohort (7 characteristics retained). **(B)** Optimized combination of 5 characteristics in the overall patient cohort. **(C)** The combination of four characteristics in the overall patient cohort. **(D)** The combination of 3 characteristics in the overall patient cohort. **(E)** Screening results of 8 characteristics in gastric stromal tumor patients (3 characteristics were retained). **(F)** Screening results of 3 characteristics in gastric stromal tumor patients (2 characteristics retained).

**Figure 2** displays the feature importance ranking of the Random Forest models. **Figures 2a–d** correspond to models for all-location GIST patients with 7, 5, 4, and 3 features, respectively. PLR emerged as the most predictive indicator with the highest mean SHAP value, significantly higher than other features (p<0.001). PIV and SII formed the second predictive echelon. Consequently, PLR remained stable across all models and was one of the most important features even when the model was refined to 3 features. **Figures 2e, f** show the feature importance ranking for gastric GIST patients with 3 and 2 features, respectively. In these model sets, PLR remained the most predictive indicator, while the importance of SII increased substantially, slightly surpassing that of PAR.

**Figure 3:**
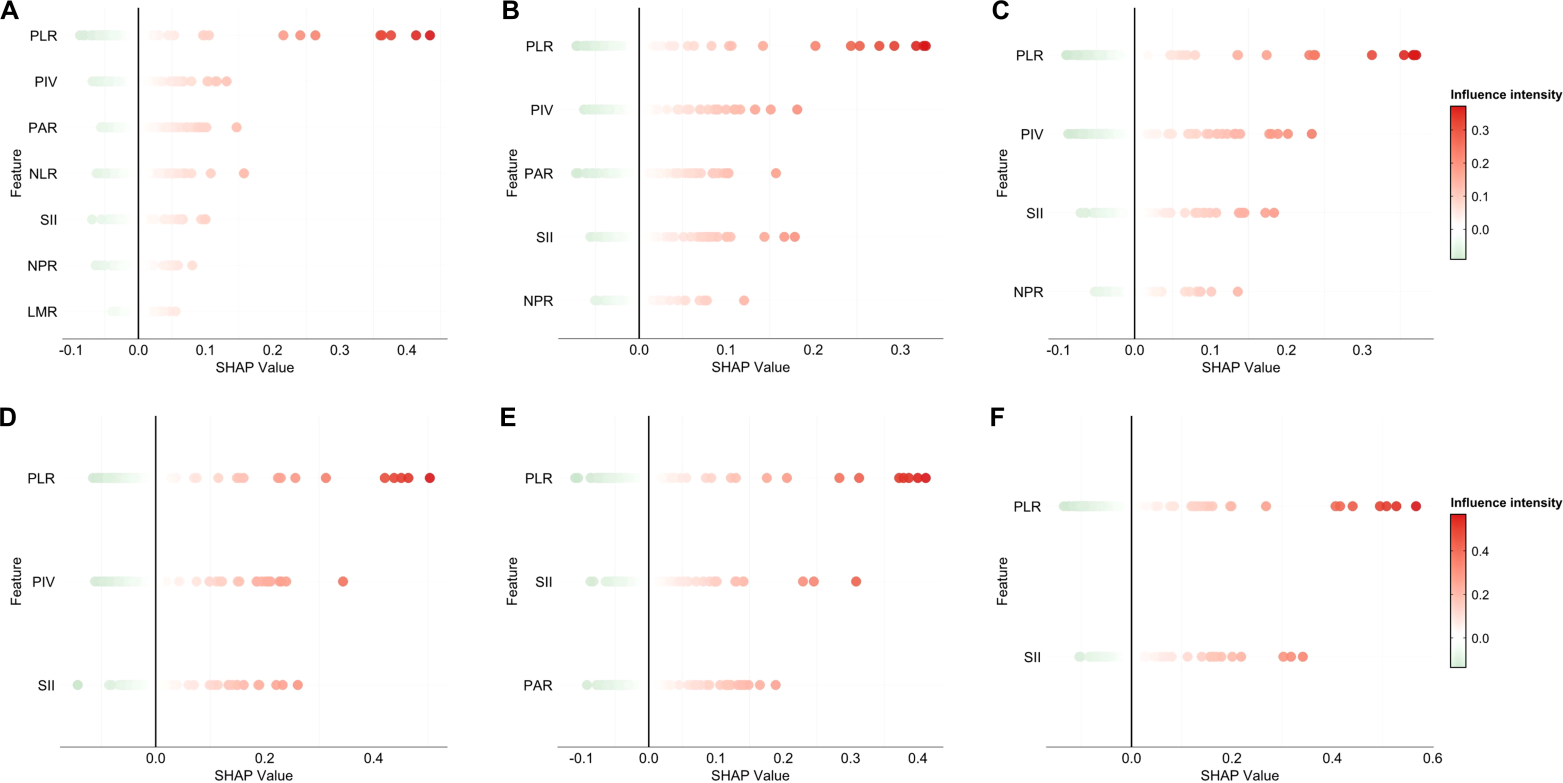
The contribution direction and intensity of the SHAP swarm graph analysis features to the recurrence risk **(A)** SHAP swarm diagram of 7 characteristic models in the whole patient cohort. **(B)** SHAP swarm diagram of 5 characteristic model for the whole patient cohort. **(C)** SHAP swarm diagram of 4 characteristic models in the whole patient cohort. **(D)** SHAP swarm diagram of 3 characteristic models in the whole patient cohort. **(E)** SHAP swarm diagram of characteristic model 3 in gastric stromal tumor patient cohort. F SHAP swarm diagram of characteristic model 2 in gastric stromal tumor patient cohort.

SHAP analysis (**Figure 3a**) further revealed: PLR had the greatest contribution, with its absolute SHAP value close to 0.4, indicating that PLR is a key positive driver for identifying high-risk recurrence. The SHAP values for SII, PIV, PAR, and NLR were primarily positive, suggesting that increases in these indicators are generally associated with higher predicted risk. The SHAP values for NPR and LMR were primarily negative, indicating that their increase is generally associated with lower predicted risk. **Figures 3b-f** are largely consistent with **Figure 3a**.

In summary, PLR, NLR, and PAR are the main factors influencing the model’s predictions, with PLR and NLR tending to increase risk, and PAR tending to decrease risk. Other factors such as SII, PIV, and NPR also have some influence, but relatively smaller. The direction of LMR’s effect differs from the others, tending to decrease risk.

### 3.5 Risk stratification of intermediate-risk patients

**Figure 4:**
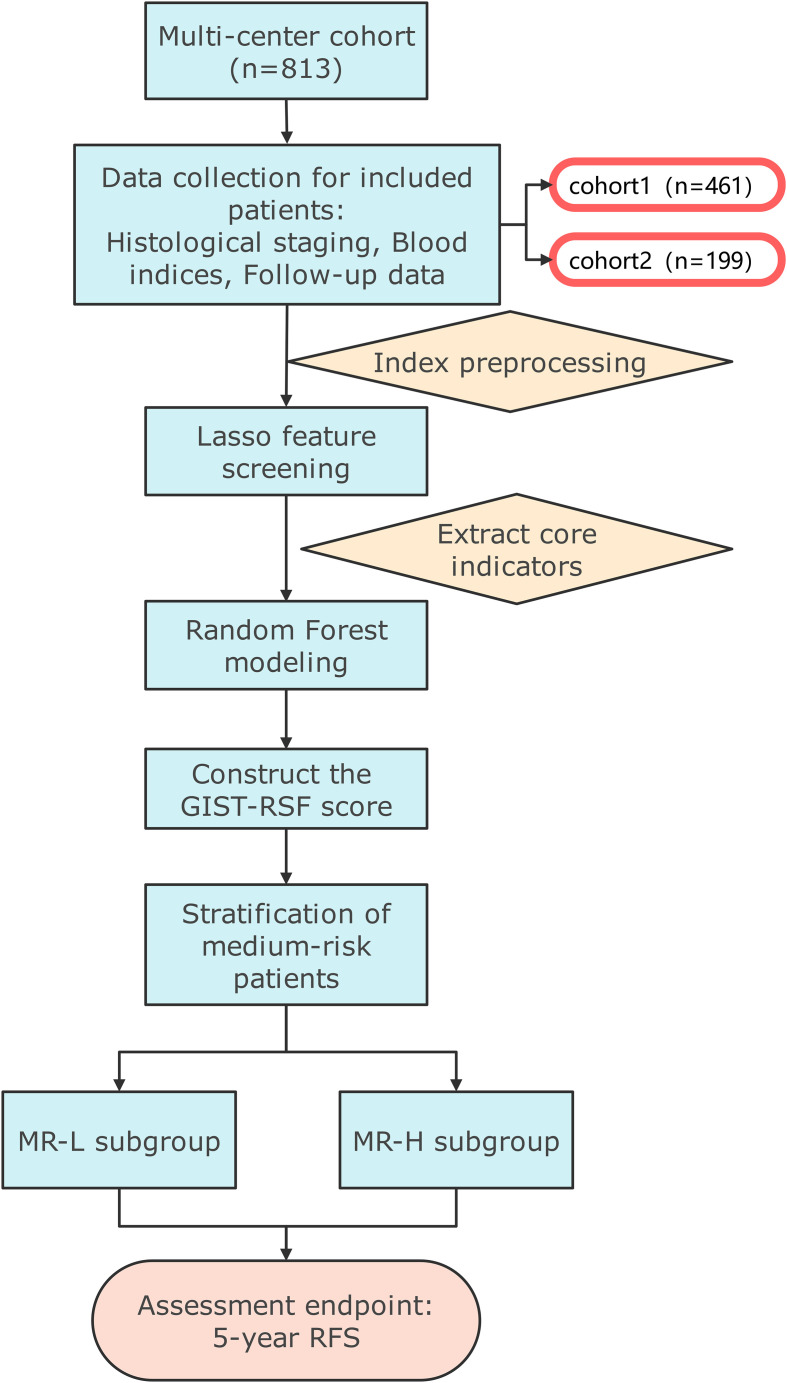
Analysis of Kaplan-Meier survival curves based on different characteristic combinations **(A-D)** The risk stratification of characteristic models was performed in the whole patient cohort 7, 5, 4 and 3 respectively. **(E, F)** Three and two characteristic models were used for risk stratification in the patient cohort of gastric stromal tumor.

Based on the predicted probabilities output by the GIST-RSF model, intermediate-risk patients were further stratified into two subgroups: “intermediate-risk low” (MR-L) and “intermediate-risk high” (MR-H). These are hereinafter referred to as the MR-L and MR-H groups, respectively. Across all models (**Figures 4a–f**), the MR-H subgroup consistently exhibited significantly poorer recurrence-free survival (RFS) than the MR-L subgroup (all p < 0.0001). For instance, in the all-location GIST model with seven features (cut-off 0.149), 32.5% of patients were classified as MR-H, showing markedly worse survival than the remaining 67.5%. Similar trends were observed across models with 5, 4, and 3 features, as well as in gastric GIST-specific models (cut-offs 0.285 and 0.290). Notably, the prognosis of the MR-L subgroup approximated that of the NIH low-risk group, whereas the MR-H subgroup resembled the NIH high-risk group. This phenomenon may be attributable to the limited sample size of the subgroup and collinearity among the indicators, suggesting the limitations of traditional regression methods in identifying independent factors within finite sample sizes.

## 4 Discussion

Our random survival forest model demonstrated excellent discrimination for recurrent risk within the intermediate-risk cohort (AUC = 0.777), and SHAP analysis particularly highlighted PLR and PAR as dominant predictive features. Through SHAP interpretability, we further corroborated that PLR and PAR represent the pivotal predictors driving the model’s outputs. This observation suggests that prognostic drivers in intermediate-risk GIST diverge from those in the broader cohort: conventional pathological metrics (tumor size, mitotic index) show inconsistent predictive utility within the intermediate subgroup, while any single inflammatory biomarker is insufficient on its own. Hence, only a composite multi-marker model can reliably stratify risk.

Our results are congruent with the GIST post-operative RFS prediction model by Jin-Long Zhao et al. incorporating SII, NLR, PLR, and MLR, further confirming the prognostic significance of inflammatory markers in GIST (14). Moreover, prior investigations have underscored that elevated PLR is significantly associated with recurrence risk in GIST patients, and functions as an independent prognostic factor for RFS (HR≈3.04) (17). Broader retrospective studies have similarly suggested that systemic inflammatory indices such as SII, PLR, and MLR may be informative in evaluating recurrence risk in GIST (18). Emerging composite metrics integrating inflammatory and nutritional status (e.g., combining SII with prognostic nutritional index, PNI) have shown promising prognostic value in locally advanced GIST patients undergoing neoadjuvant imatinib therapy, offering a novel avenue for assessing treatment response and outcome (19).

Based on the comprehensive analysis above, our GIST-RSF model successfully further stratified the traditional “intermediate-risk” population into two subgroups: MR-H (with significantly poorer prognosis, approaching that of the traditional high-risk group, p < 0.001) and MR-L (with favorable prognosis). Approximately 30% of patients with “occult high-risk” features were accurately identified, suggesting that this subgroup could serve as a candidate population for extended adjuvant therapy (e.g., beyond three years), whereas the MR-L subgroup might be spared from overtreatment—a hypothesis that warrants further validation in prospective studies. The strong correlation between early recognition and improved prognosis in severe adverse reactions such as SJS/TEN underscores the clinical value of our risk stratification model for the early identification of high-risk patients (20). Similar to the scenario in Zhao et al., where some patients continued nivolumab with controlled psoriasis, the decision to adjust adjuvant therapy duration for our identified high-risk patients requires a careful balance between therapeutic benefits and potential risks (21).

Within the intermediate-risk cohort, 58.4% (n = 90) underwent TKI therapy, whereas 41.6% (n = 64) did not. Upon risk re-stratification via the five-feature random forest, 31.1% (n = 28) of TKI-treated patients were classified as MR-H and 68.8% (n = 62) as MR-L In the non-TKI cohort, the proportions were 26.5% (n = 17) and 73.4% (n = 47), respectively. The difference in subgroup distribution between TKI and non-TKI groups was not statistically significant, suggesting that TKI exposure is unlikely to be a major confounder in influencing the model’s stratification.

Although the gastric submodel shows slightly lower AUC and sensitivity compared to the full model, its negative predictive value (NPV) and overall accuracy remain robust. Thus, it may be well suited for initial screening in primary care settings or in patients with multiple comorbidities, to “first exclude low-risk” and reduce false positives and unnecessary workups. However, given its limited sensitivity, the model is better suited as an ancillary decision-making tool, to be used in integrated assessment with other clinicopathological factors, rather than as a replacement for existing standards.

In China, high-quality medical resources are mainly concentrated in large cities, resulting in significant regional disparities and transportation burdens for patients seeking medical care (22). A blood-based surveillance strategy leveraging inflammatory markers (e.g. NLR, PLR) offers several practical advantages — easy sampling, low cost, and capacity for serial measurements. It is particularly valuable in resource-limited or remote settings, or for patients unable to undergo contrast-based imaging, as a viable alternative to radiographic surveillance (23, 24). Furthermore, its ability to dynamically monitor biomarker fluctuations and its feasibility for deployment in decentralized labs could facilitate a “home–community–hospital” coordinated follow-up model.

Notably, within the intermediate-risk subgroup, traditional multivariate Cox regression failed to identify any risk factors independently associated with RFS. This phenomenon may be attributed to the limited subgroup sample size (n=154) and the inherent collinearity among inflammatory indicators—given that PLR, NLR, and SII are all derived from the same complete blood count parameters, multivariate adjustment tends to result in variance inflation of estimates and attenuation of independent effects. However, this limitation paradoxically underscores the rationale for employing machine learning strategies in this study: ensemble algorithms such as random survival forests can automatically handle variable interactions and collinearity, effectively capturing the joint effects of multiple indicators. Thus, although individual inflammatory markers failed to maintain “independence” in traditional regression, the composite inflammatory signatures constructed from them exhibited stable predictive capacity within the machine learning framework.

This study adopted a dual-center retrospective design and employed multivariate adjustment to mitigate confounding bias. However, the limited sample size—particularly within the gastric subgroup—may affect model stability when encountering extreme values or rare mutation subtypes. Furthermore, due to the retrospective nature of the data, information regarding treatment adherence, dose intensity, and imaging review consistency was unavailable, which may introduce unmeasured bias. Importantly, this study did not incorporate treatment-related information and therefore cannot directly inform therapeutic decisions. The primary limitation of this study is the absence of an independent external validation cohort; consequently, the generalizability of the current model remains uncertain. Future multi-center prospective cohort studies, employing methods such as 1:1 propensity score matching or instrumental variable analysis, are warranted to further validate the external transportability of the model. Serial ctDNA profiling often parallels or even precedes radiographic response (25). Technologically, integrating circulating tumor DNA (ctDNA) with inflammatory biomarkers holds promise for constructing a “molecular + immune” dual-axis early screening framework, thereby providing a refined pathway for early relapse risk surveillance.

Ultimately, we advocate for a randomized controlled trial (RCT) in which intermediate-risk patients stratified by the GIST-RSF model are randomized to standard surveillance versus tailored intensified therapy arms, with overall survival (OS) and recurrence-free survival (RFS) as primary endpoints — thus directly testing whether model-driven precision strategies translate into meaningful survival gains.

## 5 Conclusion

Our GIST-RSF model effectively stratified intermediate-risk patients into two subgroups with distinct prognostic outcomes. The intermediate-risk high subgroup may represent a candidate population for extended adjuvant therapy, thereby offering a potential stratification variable for the design of future prospective studies. This study provides initial evidence that the model may facilitate individualized adjuvant treatment strategies; however, its clinical utility warrants further validation through prospective investigations.

## Data Availability

The raw data supporting the conclusions of this article will be made available by the authors, without undue reservation.
